# Enhanced recovery after liver surgery in cirrhotic patients: a systematic review and meta-analysis

**DOI:** 10.1186/s13741-024-00375-x

**Published:** 2024-04-01

**Authors:** Constant Delabays, Nicolas Demartines, Gaëtan-Romain Joliat, Emmanuel Melloul

**Affiliations:** Department of Visceral Surgery, Lausanne University Hospital CHUV, University of Lausanne (UNIL), Lausanne, Switzerland

**Keywords:** Hepatectomy, Perioperative care, ERAS, Hepatic resection, Fibrosis

## Abstract

**Background:**

Few studies have assessed enhanced recovery after surgery (ERAS) in liver surgery for cirrhotic patients. The present meta-analysis assessed the impact of ERAS pathways on outcomes after liver surgery in cirrhotic patients compared to standard care.

**Methods:**

A literature search was performed on PubMed/MEDLINE, Embase, and the Cochrane Library. Studies comparing ERAS protocols versus standard care in cirrhotic patients undergoing liver surgery were included. The primary outcome was post-operative complications, while secondary outcomes were mortality rates, length of stay (LoS), readmissions, reoperations, and liver failure rates.

**Results:**

After evaluating 41 full-text manuscripts, 5 articles totaling 646 patients were included (327 patients in the ERAS group and 319 in the non-ERAS group). Compared to non-ERAS care, ERAS patients had less risk of developing overall complications (OR 0.43, 95% CI 0.31–0.61, *p* < 0.001). Hospitalization was on average 2 days shorter for the ERAS group (mean difference − 2.04, 95% CI − 3.19 to − 0.89, *p* < 0.001). Finally, no difference was found between both groups concerning 90-day post-operative mortality and rates of reoperations, readmissions, and liver failure.

**Conclusion:**

In cirrhotic patients, ERAS protocol for liver surgery is safe and decreases post-operative complications and LoS. More randomized controlled trials are needed to confirm the results of the present analysis.

## Introduction

Liver surgery is challenging for both surgeons and anesthetists. Compared to other abdominal procedures, liver resection is at particular risk of intraoperative bleeding, massive fluid shift, transient hypotension, and a high post-operative complication rate. It is encumbered with significant overall mortality between 1 and 4%, as well as with morbidity between 40 and 50% (Song et al. 2016; Benzoni et al. 2007). Those outcomes are impacted by the extent of liver resection, surgical approach, patient comorbidities, and underlying liver disease. Cirrhosis has a systemic impact, with changes in fluid balance, coagulation, immune system, nutritional status, and many organ dysfunctions (Tsochatzis et al. 2014). Cirrhotic patients are therefore particularly vulnerable to the stress induced by both surgery and anesthesia (Agarwal and Divatia 2019). Post-operative morbidity and mortality in those patients are significantly higher, in particular after liver surgery (Csikesz et al. 2009). Belghiti et al. reported a mortality rate of 8.7% after hepatectomy in cirrhotic patients (Belghiti et al. 2000).

Enhanced recovery after surgery (ERAS) programs are standardized peri-operative protocols, designed to reduce patient’s response to surgical stress and to decrease the post-operative complication risk. Improved recovery leads to shorter lengths of stay (LoS) and reduced costs (Roulin et al. 2013). Those protocols incorporate a panel of recommendations based on a multimodal approach and have demonstrated safety and efficacity in several types of surgery, especially in colorectal surgery (Greco et al. 2014; Labgaa et al. 2016; Modesitt et al. 2016). Implementation of those protocols needs a strong collaboration between surgeons, anesthetists, nurses, physiotherapists, and also patients. ERAS concepts in liver surgery were first reported in 2008 by Van Dam et al. and proven to be safe and effective with shortened LoS (Dam et al. 2008). Since then, many studies have demonstrated the positive impact of ERAS pathways among patients undergoing liver resection: shorter LoS, decreased morbidity, reduced costs and biological stress, and increased patient satisfaction, without changes in readmission rate or mortality (Noba et al. 2020; Joliat et al. 2016). In 2016, the ERAS society published the first guidelines for liver surgery in an effort to bring harmonization to various protocols (Melloul et al. 2016). These guidelines were published without specific consideration for cirrhotic patients since data on these particularly vulnerable patients was lacking at that time. Only a few studies have assessed the applicability and impact of ERAS programs in cirrhotic patients undergoing liver surgery (Lunel et al. 2021).

The aim of the present meta-analysis was to assess the impact of ERAS pathways on outcomes after liver surgery in patients with cirrhosis compared to standard care.

## Methods

### Search strategy

This study follows the recommendations of the PRISMA (Preferred Reporting Items for Systematic Reviews and Meta-Analyses) guidelines (Moher et al. 2009). Publications were selected by searching PubMed/MEDLINE, Embase, and the Cochrane Library. The search was performed independently by 2 researchers (CD and GRJ) and ended on 1st February 2022. The search terms to find eligible articles were: enhanced recovery after surgery (MeSH term), enhanced recovery pathway (free text), fast track surgery (free text), liver surgery (free text), liver resection (free text), hepatocellular carcinoma (free text), liver cancer (free text), hepatectomy (MeSH term), and liver cirrhosis (MeSH term). All these MeSH and free text terms were combined with “OR” or “AND”. All titles were reviewed, and if relevant, abstracts were subsequently analyzed. If the abstract was of interest, full texts were then reviewed. References of the selected articles were also browsed (cross-referencing). Ethical approval was not necessary because this study was a systematic review and meta-analysis.

### Inclusion and exclusion criteria

Only studies that compared the ERAS program to the non-ERAS program in cirrhotic patients with liver resection were included in the meta-analysis. If a study included cirrhotic patients as a subgroup analysis, the article was also included. In retrospective studies, only comparative studies with more than 40 cirrhotic patients were included for meta-analysis. The description of an enhanced recovery pathway was mandatory.

### Data extraction

The following information was extracted by 2 researchers (CD and GRJ) from each of the studies: first author’s name, publication year, country, study type, operative approach, total number of cases, demographics, outcome measures, ERAS protocol, discharge criteria, and study quality.

### Outcomes of interest

Outcomes of interest were the occurrence of any complication, mortality rate, LoS, need for readmission and for reoperation, and occurrence of liver failure. Complications were assessed according to the Clavien classification (Dindo et al. 2004). Mortality and complications were evaluated within 90 days post-operatively. LoS was defined as the number of days from the day of surgery until discharge.

### Quality assessment

The quality of studies was assessed according to the Cochrane Collaboration tool for assessing the risk of bias (Higgins et al. 2011). The following criteria were analyzed: random sequence generation (for randomized control trial, RCT), allocation concealment (for RCT), blinding of participants and personnel (for RCT), blinding of outcome assessment (for RCT), incomplete outcome data, selective reporting, and other bias.

### Statistical analysis

All statistical analyses were performed using Review Manager (RevMan) software, version 5.4, from the Cochrane collaboration (Manager and (RevMan) [Computer program]. The Cochrane Collaboration 2020). Odds ratios (OR) with the Mantel-Haenszel method were used for the statistical comparison of dichotomous data, while mean differences (MD) with inverse variance methods were applied for continuous variables. A 95% confidence interval (CI) was reported for both OR and MD. *I*^2^ values were used for quantification of statistical inconsistency, defined as the percentage of variation between included studies due to heterogeneity (Higgins et al. 2003). To take into account clinical heterogeneity, a random effect model was used for the meta-analyses. An I^2^ value exceeding 50% was considered a significant heterogeneity. If the study provided medians and ranges instead of mean and standard deviation, the latter were calculated as described by Hozo et al. (Hozo et al. 2005). Forest plots were built using the random effect model assuming that the true effect size varies between studies and a *p* value < 0.05 was considered to represent statistical significance.

## Results

### Eligible studies

**Fig. 1:**
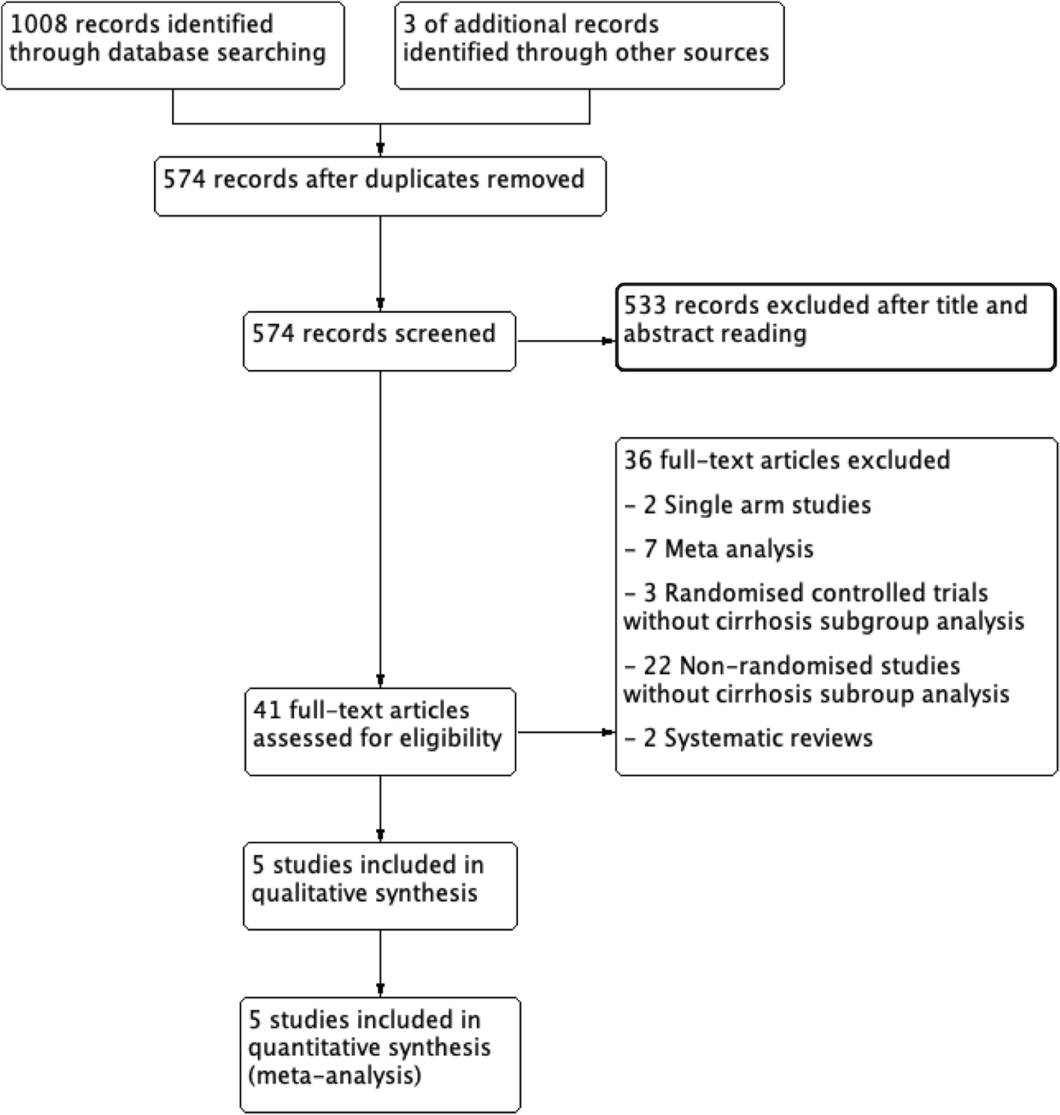
PRISMA flow chart of the study

A total of 1011 studies were initially identified. The study flow chart is shown in Fig. 1. After duplicate removal, titles and abstracts of 574 studies were reviewed. Among them, 41 articles comparing ERAS *vs*. non-ERAS protocols for liver surgery were identified. Many of those studies included cirrhotic patients, but only five studies included cirrhotic patients only or had a subgroup analysis of cirrhotic patients, and could be included in the meta-analysis (Lunel et al. 2021; Gonvers et al. 2021; Qi et al. 2018; Zheng et al. 2020; Zhou et al. 2021). If the terms “liver cancer” or “hepatocellular carcinoma” were taken into account in the search equation, no further article to include was found.

### Study characteristics

The characteristics of the included studies are presented in Table 1. Only 1 study was RCT, and the others were observational studies with historical comparison groups of non-ERAS patients before the implementation of ERAS protocols. Three of four observational studies had prospective data collection. The surgical approach was laparoscopic and open liver resection for 4 studies (Lunel et al. 2021; Gonvers et al. 2021; Qi et al. 2018; Zheng et al. 2020), and laparoscopic only for 1 study (Zhou et al. 2021). The 5 studies included a total of 1467 patients. Among those patients, only cirrhotic patients were considered, totaling 646 patients: 327 in the ERAS group and 319 in the non-ERAS group. All studies described their ERAS protocols (Table 2). Three studies followed the 2016 ERAS recommendations, while 2 had their own homemade ERAS protocols. The latter were however very similar to guidelines. The overall compliance rate was specified in one study only and was 60% (Lunel et al. 2021). Child scores were specified in 3 out of 5 studies (Lunel et al. 2021; Qi et al. 2018; Zheng et al. 2020). The rates of Child A patients were very variable (Lunel et al.: 95%, Qi et al.: 13%, and Zheng et al.: 100%), but similar between ERAS and control groups among all three studies. Rates of major hepatectomy were similar in each study among ERAS and control groups. Etiologies of cirrhosis were fully specified in only 1 study and partially provided in two others, but none performed any subgroup analysis (Lunel et al. 2021; Zheng et al. 2020; Zhou et al. 2021). Lunel et al. present a heterogeneous population: 52 alcohol-induced cirrhosis, 22 hepatitis C virus (HCV) cirrhosis, 13 patients with hepatitis B virus (HBV), 37 patients with nonalcoholic steatohepatitis (NASH), and 2 patients with other causes. Zhou et al. present 149 patients out of 174 with HBV-induced cirrhosis and Qi et al. present 50 patients out of 180 with HBV-induced cirrhosis (Qi et al. 2018; Zhou et al. 2021). Hepatocellular carcinoma (HCC) was the main indication for surgery (416 out of 646 patients, 64.4%), and two studies included only HCC patients (Zheng et al. 2020; Zhou et al. 2021). Other indications were heterogeneous and are specified in Table 1.

**Table 1:** Main characteristics of the included studies (*n* = 5)

Study	Country	Design	Type of surgery	Total n of patients	Cirrhotic patients		Age (y, median)		Men, n (%)		CPT A, n (%)		Diagnosis	Major hepatectomy, n (%)	
					ERAS	CTL	ERAS	CTL	ERAS	CTL	ERAS	CTL		ERAS	CTL
Gonvers et al. 2020	Switzerland	NRS	Lap. and open	541	21 (47%)	24 (53%)	64	64.5	17 (81)	21 (87)	N/a	N/a	HCC: 32 (71) Other: 13 (29)	7 (33)	11 (46)
Lunel et al. 2021	France	NRS	Lap. and open	430	59 (56%)	46 (44%)	67	65	54 (91.5)	44 (95.7)	55 (93)	45 (98)	HCC: 96 (91) Other: 9 (9)	14 (24)	9 (19)
Qi et al. 2018	China	RCT	Lap. and open	160	80 (50%)	80 (50%)	53.7	55.4	47 (58.8%)	40 (50)	8 (10)	13 (16)	Liver tumor^:^ 68 (43) Other: 92 (57)	28 (35)	31 (39)
Zheng et al. 2020	China	NRS	Lap. and open	114	80 (49%)	82 (51%)	55	59	64 (80)	59 (72)	80 (100)	82 (100)	HCC: 114 (100)	30 (38)	21 (26)
Zhou et al. 2021	China	NRS	Lap.	174	87 (50%)	87 (50%)	54.9	55.2	66 (75.9)	70 (80.5)	N/a	N/a	HCC: 174 (100)	5 (6)	1 (1)

*NRS* Non-randomized study, *RCT* Randomized clinical trial, *Lap* laparoscopic, *CTL* Control, *CPT* Child-Turcotte-Pugh, *N/a* Not available, *HCC* Hepatocellular carcinoma. Diagnoses are given to cirrhotic patients only. Other diagnoses were hepatolithiasis (*n* = 92), Colorectal metastasis (*n* = 5), adenoma *n* = 2), hepatic cyst (*n* = 1), hemangioma (*n* = 1), Caroli disease (*n* = 1), micronodular cirrhosis (*n* = 1), right liver atrophy (*n* = 1), not specified (*n* = 9). Qi et al. did not specify the type of liver tumor

**Table 2:** ERAS Items among studies

ERAS Items according to 2016 Guidelines		Gonvers et al.	Lunel et al.	Qi et al.	Zheng et al.	Zhou et al.
Preoperative	Perioperative education	Yes	Yes	Yes	Yes	Yes
	Perioperative nutrition for risk patients	Yes	Yes	Yes	No	Yes
	Carbohydrate loading 2 h before surgery. Preoperative fasting does not exceed 6h for solid, and 2h for liquids	Yes	Yes	Yes	Yes	Yes
	No bowel preparation	Yes	Yes	Yes	Yes	Yes
	LAAD avoided	Yes	Yes	No	No	No
	LMWH 12 after hepatectomy	Yes	Yes	No	No	Yes
	Steroid used before operation	Yes	Yes	No	No	Yes
	Antibiotics used 1 h before hepatectomy	Yes	Yes	No	Yes	Yes
	PONV prophylaxis, and multimodal	Yes	Yes	No	Yes	Yes
Intraoperative	Mercedes incision avoided	Yes	Yes	No	No	No
	Minimal invasive approach when possible	Yes	Yes	Yes	Yes	Yes
	No systematic gastric tube	Yes	Yes	No	Yes	Yes
	Perioperative normothermia	Yes	Yes	No	Yes	Yes
	Drain as little as possible	Yes	Yes	Yes	No	Yes
	Omental flap to against delay gastric empty	Yes	Yes	No	No	No
	Goal-directed fluid therapy with low CVP	Yes	Yes	Yes	Yes	Yes
	Crystalloids are preferred over 0.9% saline and colloids	Yes	Yes	No	No	No
Post-operative	Early oral intake	Yes	Yes	Yes	Yes	Yes
	Glycemic control	Yes	Yes	No	Yes	No
	No stimulation of transit	Yes	Yes	No	No	No
	Early mobilization	Yes	Yes	Yes	Yes	Yes
	Multimodal analgesia	Yes	Yes	Yes	Yes	Yes

*LAAD* long-acting anxiolytic drugs should, *LMHW* Low molecular weight heparin, *PONV* Post-operative nausea and vomiting, *CVP* Central venous pressure

### Quality assessment

**Fig. 2:**
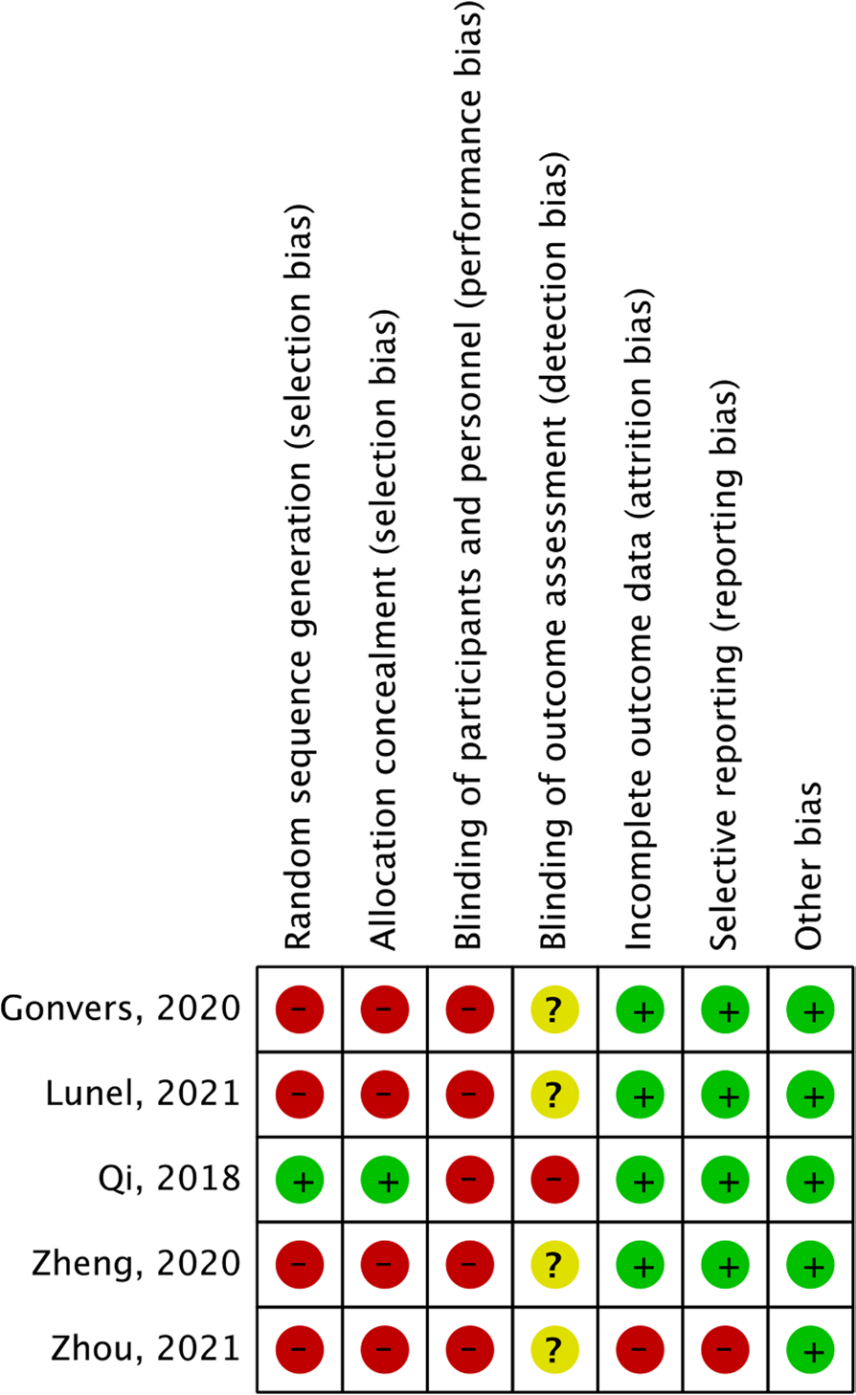
Quality assessment using the Cochrane Collaboration tool. Risk of bias summary in controlled trials. The symbol (−) indicates that there is a high risk of bias, (+) indicates a low risk of bias, and (?) indicates uncertainty

The quality of all five studies is summarized and represented in Fig. 2. The assessment was discussed between two of the authors (CD and GRJ) until a consensus was reached. Only one of the studies was an RCT and therefore had a low risk of bias for random selection bias (Qi et al. 2018). Blinding of participants was not applicable because of the multimodal approach of ERAS and the importance of education. In concern with the other biases, the included studies were of good quality.

### Post-operative complications

**Fig. 3:**
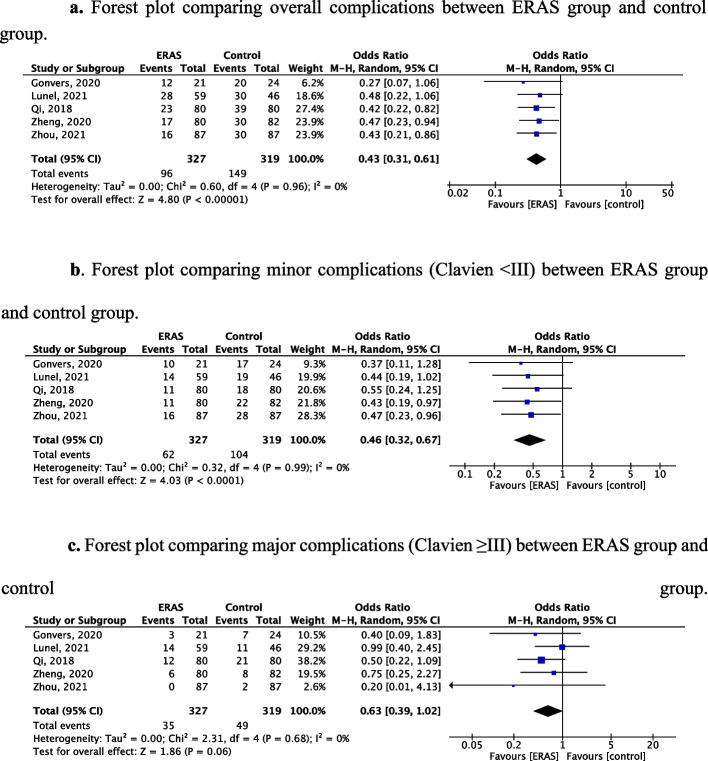
Forest plots comparing complications between ERAS and the control group. **a** Forest plot comparing overall complications between the ERAS group and control group. **b** Forest plot comparing minor complications (Clavien < III) between ERAS group and the control group. **c** Forest plot comparing major complications (Clavien ≥ III) between ERAS group and control group

**Fig. 4:**
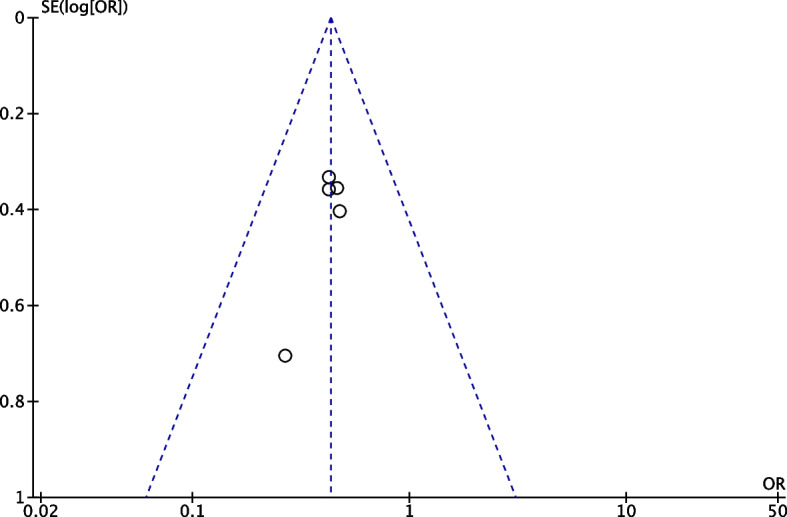
Funnel plot for risk of publication bias regarding overall post-operative complications

All studies reported post-operative morbidity using the Clavien classification. Compared to standard care, cirrhotic patients following ERAS protocol had about 57% less probability of developing any complication (OR 0.43, 95% CI 0.31–0.61, *p* < 0.00001, Fig. 3a). When comparing only minor complications (Clavien < III), similar results were found with a significantly lower rate of minor complication for ERAS patients (OR 0.48, 95% CI 0.31–0.72, *p* = 0.0005, Fig. 3b). The rates of major complications were similar in both groups (OR 0.63, 95% CI 0.39–1.02,* p* = 0.06, Fig. 3c). No publication bias was found on the funnel plot (Fig. 4). Post-operative ascites were specified in one study (Zheng et al. 2020) and were significantly lower among ERAS patients (3 *vs*. 12 patients, *p* = 0.046).

### days post-operative mortality

**Fig. 5:**
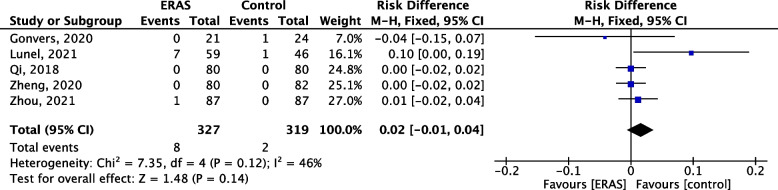
Forest plot comparing post-operative mortality between the ERAS group and control group

All studies reported mortality. There was no statistical difference between ERAS and non-ERAS groups in terms of mortality (risk difference 0.02, 95% CI 0.01–0.04, *p* = 0.14, *I*^2^ = 46%, Fig. 5). Overall mortality was 1.54% (10/646).

### Length of stay

**Fig. 6:**
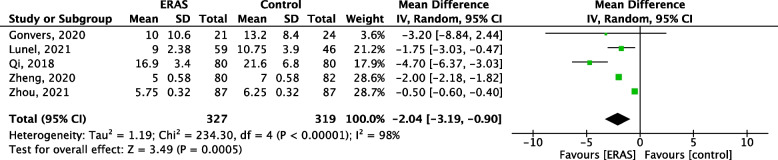
Forest plot comparing the length of stay between the ERAS group and the control group

All 5 studies reported LoS. Among them, 3 were expressed as median (Lunel et al. 2021; Zheng et al. 2020; Zhou et al. 2021). Means and standard deviations were therefore calculated as described by Hozo et al. (Hozo et al. 2005). The ERAS group (*n* = 327) stayed significantly less long in the hospital than control (*n* = 317). Mean differences was 2.04 days (CI − 2.04, − 0.89) in favor of patients following ERAS protocols (*p* < 0.00001, Fig. 6). Discharge criteria were similar between studies.

### Hospital readmission

**Fig. 7:**
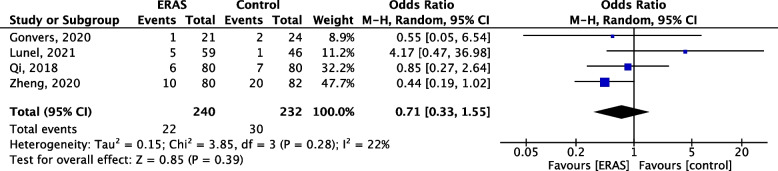
Forest plot comparing the need for readmission between the ERAS group and control group

All trials except one (Zhou et al. 2021) reported hospital readmission rate after discharge (*n* = 472). Timing to report readmission was however variable (90 days for Lunel et al. and Gonvers et al. 30 days for Qi et al. and no information for Zheng et al.). No significant statistical difference between both groups (OR 0.7, 95% CI 0.33–1.55, *p* = 0.39) was observed. Heterogeneity was low (*I*^2^ = 22%, Fig. 7).

### Need for reoperation

**Fig. 8:**
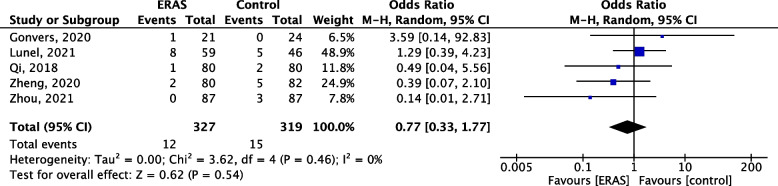
Forest plot comparing the need for reoperation between the ERAS group and control group

All included studies reported rates of reoperation, sometimes referred to as grade IIIb complication using the Clavien classification. When comparing ERAS to non-ERAS cirrhotic patients, similar reoperation rates were displayed (OR = 0.77, 95% CI 0.33–1.77, *p* = 0.54). *I*^2^ was 0%, indicating low heterogeneity between studies (Fig. 8).

### Liver failure

Only 3 studies reported rates of post-operative liver failure (*n* = 427) (Lunel et al. 2021; Qi et al. 2018; Zheng et al. 2020). Both ERAS and non-ERAS groups had similar liver failure rates (OR 1.17, 95% CI 0.47–2.78, *p* = 0.53, *I*^2^ = 0%, Fig. 8). The overall incidence of liver failure among both groups was 5.2% (22/427). Liver failure was, however, clearly defined in only one study (Lunel et al. 2021) Fig. 9.

**Fig. 9:**
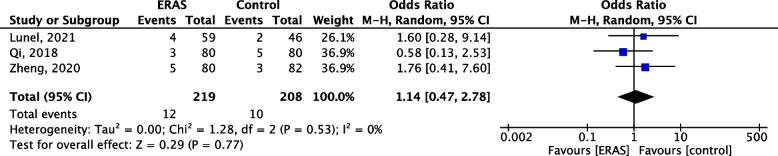
Forest plot comparing post-operative liver failure between the ERAS group and control group

## Discussion

This is the first meta-analysis that assesses the impact of ERAS protocols on outcomes after liver resection in cirrhotic patients. The results of this present study demonstrated the safety (same mortality rate) and benefits in terms of post-operative complications (OR = 0.43, 95% CI 0.31–0.61) of ERAS programs in cirrhotic patients compared to standard care. This is of importance, as cirrhotic patients are fragile and should not be excluded from the ERAS pathway.

ERAS programs are widely applied, and many studies have assessed the positive impact of those protocols for liver surgery: shorter LoS, decreased morbidity and cost, increased patient satisfaction and pain control, with no mortality or readmission increase (Song et al. 2016; Yang et al. 2016; Wang et al. 2017). However, cirrhotic patients were not assessed as a group in those ERAS studies. In fact, cirrhotic patients were either excluded or mixed with non-cirrhotic patients, making specific interpretation impossible. In addition, the 2016 ERAS guidelines for liver resection were developed without specific attention to cirrhotic patients. Systemic pathophysiological changes induced by cirrhosis make these patients more vulnerable to surgical stress. The benefits of ERAS in this population remain unclear. Lunel et al. reported that ERAS guidelines were not deleterious for cirrhotic patients undergoing hepatectomy, but they did not show benefits either (Lunel et al. 2021). Zheng et al*.* reported a positive impact of ERAS protocols with decreased LoS and reduced post-operative complications (Zheng et al. 2020). The present meta-analysis demonstrated a positive impact of ERAS programs on overall morbidity and LoS, without any effect on mortality or readmission rate.

Compliance is a major determinant of the efficacity of ERAS protocols. Based on various data, the International ERAS Society suggests that 70% compliance is the threshold to observe the positive effect of the ERAS program on morbidity. Compliance with ERAS items was specified in only one study (Lunel et al. 2021). In that trial, the authors reported a benefit of ERAS despite an overall compliance rate of 60%. Of note, compliance rates were similar in both groups (cirrhotic and non-cirrhotic patients).

Significant progresses have been made in preoperative assessment of liver function and precise operative planification. Due to a historical lack of clear definition, the reported incidence of post-hepatectomy liver failure (PHLF) is very variable in the literature and ranges from 1 to 37% (Ray et al. 2018). The International Study Group of Liver Surgery (ISGLS) established the first internationally standardized definition and grading of PHLF in 2011 (Reissfelder et al. 2011). Definitions were then extended to biliary leak and hemorrhage in order to standardize specific post-hepatectomy complications (Rahbari et al. 2011 Rahbari et al. 2011). PHLF was clearly stated in only one study and was defined as a bilirubin level >120 μmol/l on any post-operative day (Lunel et al. 2021). No difference in PHLV was found in ERAS vs non-ERAS groups. Impacts on other specific post-hepatectomy complications were not analyzed due to the lack of data and standardized definitions in the different studies. ERAS protocols tend to reduce low-grade and medical complications and have less impact on severe post-operative complications (Song et al. 2016; Ni et al. 2015). We may therefore expect a limited impact of ERAS protocol on biliary leak and hemorrhage as already shown in a recent meta-analysis (Li et al. 2017). A meticulous preoperative assessment of liver function and tumor extent to apply a parenchyma-sparing strategy is paramount for cirrhotic patients. More studies are needed with clear and standardized definitions such as those proposed by the IGSLS to better evaluate ERAS impact on specific post-hepatectomy complications.

Due to the specific and systemic pathological changes among cirrhotic patients, the application of several items from ERAS guidelines may not be suitable for those patients. Cirrhotic patients could therefore benefit from specific adapted recommendations. Pain control is one of the key elements in ERAS pathways. Thoracic epidural analgesia (TEA) is sometimes used after liver surgery to facilitate early mobilization, and therefore decreasing respiratory and thromboembolic complications (Reissfelder et al. 2011). However, TEA can be associated with hypotension, and there is concern about the safety of catheter removal due to post-operative coagulopathy (Sakowska et al. 2009). The development of coagulopathy could delay catheter removal and necessitate the need for fresh frozen plasma. Moreover, one RCT showed that TEA could be a risk factor for post-operative renal failure due to hypotension (Rahbari et al. 2011). This concern is even more important among cirrhotic patients due to preexisting coagulopathy and their fluid balance. However, two studies evaluated TEA among cirrhotic patients undergoing liver resection and did not find any complications related to epidural catheter placement or removal (Siniscalchi et al. 2016; Esteve et al. 2017). Patients with TEA had a shorter length of mechanical ventilation and LoS (Siniscalchi et al. 2016). Even if TEA showed better pain control, wound catheters showed similar post-operative outcomes in a meta-analysis of 4 studies (Bell et al. 2019). Incisional catheters could therefore be a good alternative for cirrhotic patients. However, collateral abdominal wall veins during catheter placement may be challenging. Intravenous patient-controlled analgesia (IV PCA) has also shown good results among cirrhotic patients when compared to TEA (Fayed et al. 2014).

ERAS guidelines recommended the use of an anti-thrombotic prophylaxis, which should be promptly started after intervention. Liver resection itself is associated with increased thrombotic risk and advocates more aggressive antithrombotic prophylaxis (Melloul et al. 2012). In cirrhotic patients, there is a complex and dynamic imbalance between procoagulant and anticoagulant factors. International normalized ratio (INR) and activated partial thromboplastin time (aPTT) only evaluate the lack of procoagulant factors and do not consider the drop of anticoagulant factors like protein C and S and the relative increase of von Willebrand factor. A recent study even showed that prolonged INR and aPTT were related to increased prothrombotic risk without increased bleeding risk (Zermatten et al. 2020). Conventional tests for coagulation should therefore be interpreted with caution among cirrhotic patients. Viscoelastic coagulation tests could offer a better evaluation of coagulation balance (Mallett et al. 2016). In a survey from 2014, 35% of surgeons reported waiting until no biological sign of coagulopathy before starting thromboprophylaxis (Weiss et al. 2014). Among the included studies, Lunel et al. reported a very low compliance rate for thromboprophylaxis (< 10%) and did not start prophylactic anticoagulation before ruling out post-operative coagulopathy. Two studies did not report the use of thrombotic prophylaxis in their ERAS program (Zheng et al. 2020; Zhou et al. 2021). The rates of thromboembolic events were also not reported in all included studies.

Malnutrition is a leading modifiable risk factor for post-operative complications after major surgery. This condition is very common in chronic liver disease and is observed in 65 to 85% of cirrhotic patients (Maharshi et al. 2015). It is secondary to malabsorption, inadequate dietary intake, and altered metabolism. The 2016 ERAS and ESPEN (guidelines recommended perioperative nutrition whenever the patient presented weight loss > 10–15% within 6 months, body-mass index < 18 kg/m^2,^ or an albumin level inferior to 30 g/l (Melloul et al. 2016; Weimann et al. 2017). Those indicators are, however, not ideal for cirrhotic patients: weight is not reliable because of the possible presence of ascites and edema, and albumin levels are chronically low due to synthesis diminution. Specific tools for cirrhosis have been recently developed and could be of better use for malnutrition screening in those patients. The Royal Free Hospital Nutritional Prioritization Tool (RFH-NPT) was specifically developed for cirrhotic patients and showed a better value in predicting malnutrition risk (Morgan et al. 2006; Wu et al. 2002). The Liver Disease Undernutrition Screening Tool (LDUST) is another validated score based on six subjective questions (Booi et al. 2015). The KIRRHOS study demonstrated that RFH-NTP and LDUST were the most accurate for cirrhotic patients among eight screening tools (Georgiou et al. 2019). Further studies are needed for their application in liver surgery and the prediction of post-operative outcomes. Finally, available literature shows that nutrition support is beneficial to cirrhotic patients undergoing liver surgery (Nutritional support for liver disease - Koretz RL. 2012. Specialized regimens such as immune-enhancing diets or branched-chain amino acid-enriched nutrition have not shown superior outcomes regarding post-operative morbidity and mortality, and are therefore not recommended (Bischoff et al. 2020). Moreover, high caloric (30–35 kcal/kg/day) and protein (1.2–1.5 g/kg/day) intake should be aimed (Bischoff et al. 2020).

Some limitations of the present study have to be discussed. Among all included studies, only one trial was RCT. Including retrospective studies in a meta-analysis may induce some biases, but represent nevertheless the best currently available evidence. Since ERAS has now become the standard of care, most studies are comparing the ERAS group with a historic pre-ERAS group, which explains why most of the selected studies are non-randomized. Moreover, there were differences between ERAS protocols in the various studies, such as the systematic use of TEA, different nutritional assessment tools, and timing for thromboprophylaxis, which can limit the interpretation of the meta-analysis results. Due to limited data, subgroup analyses based on the etiology of cirrhosis or the hepatectomy extent were not feasible.

To conclude, it is suggested that the ERAS program for liver resection is safe and effective in cirrhotic patients. It shortened LoS and significantly decreased post-operative morbidity, with no increase in mortality, readmission or reoperation rates, and incidence of liver failure. As specific pathophysiological changes occur in cirrhotic patients, the next step would be to develop specific items of ERAS protocols to further improve the management of cirrhotic patients.

## Data Availability

The datasets used and/or analyzed during the current study are available from the corresponding author on reasonable request.

## Abbreviations

ERAS: Enhanced recovery after surgery
LoS: Length of stay
RCT: Randomized controlled trial
RevMan: Review Manager
OR: Odds ratios
MD: Mean differences
CI: Confidence interval
HCV: Hepatitis C virus
HBV: Hepatitis B virus
NASH: Nonalcoholic steatohepatitis
HCC: Hepatocellular carcinoma
PHLF: Post-hepatectomy liver failure
TEA: Thoracic epidural analgesia
IV PCA: Intravenous patient-controlled analgesia
INR: International normalized ratio
aPTT: Activated partial thromboplastin time
ESPEN: European Society for clinical nutrition and metabolism
RFH-NPT: Royal Free Hospital Nutritional Prioritization Tool
LDUST: Liver disease undernutrition screening tool
CPT: Child-Turcotte-Pugh
